# Biological functions, mechanisms, and clinical significance of circular RNA in pancreatic cancer: a promising rising star

**DOI:** 10.1186/s13578-022-00833-3

**Published:** 2022-06-21

**Authors:** Qun Chen, Jiajia Li, Peng Shen, Hao Yuan, Jie Yin, Wanli Ge, Wujun Wang, Guangbin Chen, Taoyue Yang, Bin Xiao, Yi Miao, Zipeng Lu, Pengfei Wu, Kuirong Jiang

**Affiliations:** 1 Pancreas Center, The First Affiliated Hospital of Nanjing Medical University, Nanjing, China; 2 Affiliated Hospital of Yangzhou University, Yangzhou, China; 3 Nanjing Hospital of Chinese Medicine, Affiliated to Nanjing University of Chinese Medicine, Nanjing, China

**Keywords:** CircRNAs, Pancreatic cancer, Biomarker, Cancer diagnosis and therapy

## Abstract

Pancreatic cancer (PC) is a highly malignant solid tumor with insidious onset and easy early metastasis. Despite tremendous efforts devoted to research in this field, the mechanisms underlying PC tumorigenesis and progression remain unclear. Additionally, robust biomarkers and satisfactory therapeutic strategies for clinical use in PC patients are still lacking. Circular RNAs (circRNAs) are a new type of non-coding RNA originating from precursor messenger RNAs, with a covalent continuous closed-loop structure, strong stability and high specificity. Accumulating evidence suggests that circRNAs may participate in PC development and progression. Abnormal expression of circRNAs in PC is considered a vital factor that affects tumor cell proliferation, migration, invasion, apoptosis, angiogenesis and drug resistance. In this review of relevant articles published in recent years, we describe the basic knowledge concerning circRNAs, including their classification, biogenesis, functions and research approaches. Moreover, the biological roles and clinical significance of circRNAs related to PC are discussed. Finally, we note the questions remaining from recent studies and anticipate that further investigations will address these gaps in knowledge in this field. In conclusion, we expect to provide insights into circRNAs as potential targets for specific PC diagnosis and treatment in the future.

## Background

Pancreatic cancer (PC) is the fourth leading cause of cancer-related death in the United States and the seventh leading cause of cancer-related death worldwide. Globally, nearly five hundred thousand new cases are diagnosed each year, which is almost equal to the number of deaths caused by PC, and the 5-year survival rate is approximately 10% [1, 2]. At present, surgical resection is the only curative treatment option available for patients with PC. However, only 10 ~ 20% of patients are diagnosed with a resectable disease, and the 5-year survival rate remains relatively low [3]. Although a few efforts in recent years have partially improved the efficacy of surgery and chemoradiotherapy, as exemplified by the application of a newer gemcitabine (GEM)-based adjuvant chemotherapy regimen referred to as modified Folfirinox (mFolfirinox), there is still a lack of robust biomarkers and effective therapeutic strategies for clinical use in PC [4].

In recent years, whole genome/exome and RNA sequencing have revealed extensive heterogeneity in PC. An increasing number of studies have implicated molecular substrates [circular RNAs (circRNAs)] as important mechanisms in PC occurrence and development and as biomarkers for early diagnosis and PC targeted therapeutic strategies. In this manuscript, we systematically reviewed the relevant articles published in recent years; summarized circRNA biogenesis, functions and research approaches; and further discuss the biological roles and clinical significance of circRNAs in PC in detail. We hope this review will present reliable evidence of the potentially important role of circRNAs in the specific diagnosis and treatment of PC in the future.

## An overview of circRNAs

CircRNAs were firstly identified as viroids in RNA viruses in 1976 and were not observed in eukaryotes until 1991, at which time there were identified as a group of single-stranded, closed-loop RNA molecules that lack terminal 5′ and 3′ ends [5, 6]. However, they were regarded as accidental byproducts of splicing errors and did not receive extensive attention until in 2013, when Jeck WR identified two mechanisms of circRNA formation: lariat-driven circularization and intron-pairing-driven circularization [7]. As sequencing and bioinformatics technologies have progressed, the properties and diverse activities of circRNAs have been revealed. CircRNAs are expressed specifically in different cell types, tissues and developmental stages [8] and are involved in various physiological and pathological conditions, such as cardiovascular diseases, diabetes and neurological disorders [9, 10]. Moreover, recent studies have revealed that circRNAs are involved in the initiation and progression of tumors and might function as prognostic biomarkers and novel therapeutic targets [11].

## CircRNA classifications and biogenesis mechanisms

**Fig. 1:**
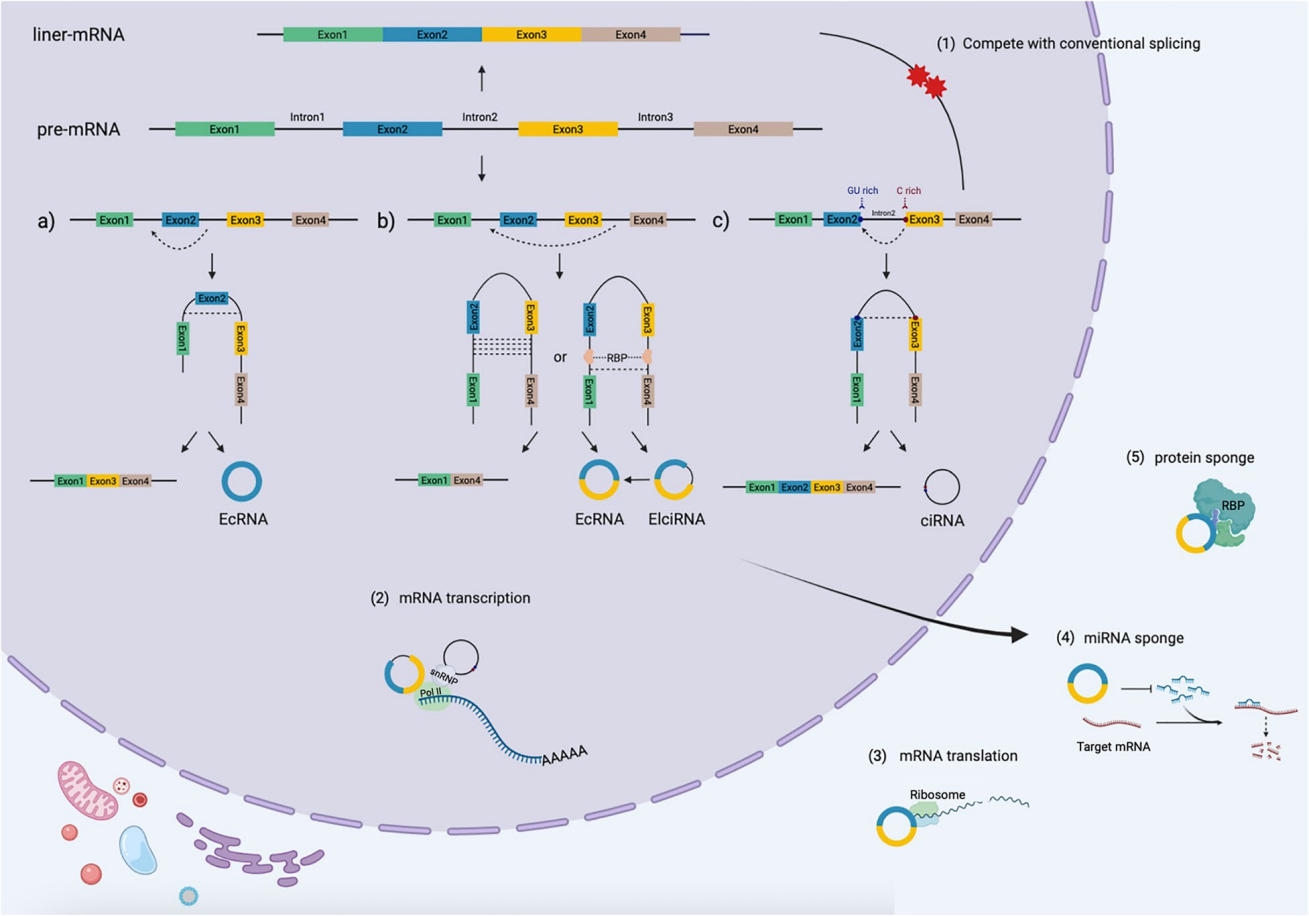
Biogenesis mechanism and functions of circular RNAs (circRNAs). Biogenesis of circRNAs. CircRNAs are generated from the 5’ splice donor site and 3’ splice acceptor site of precursor mRNAs (pre-mRNAs), which are covalently linked in reverse order. Lariat-driven circularization and intron-pairing-driven circularization are common models of circRNA formation. Additionally, some RNA binding proteins (RBPs) might act as regulatory activators or inhibitors in circRNA biogenesis. In terms of type, circRNAs can be sorted into three main categories: a) exonic circRNAs (EcRNAs), b) exon–intron circRNAs (EIciRNAs), and c) circular intronic RNAs (ciRNAs). The functions of circRNAs are as follows: (1) to compete with conventional splicing, (2) to act as transcriptional modulators, (3) to serve as translation templates of proteins, (4) to act as competing endogenous RNAs (ceRNAs), and (5) to bind to proteins

Unlike the canonical splicing of mRNAs, circRNAs are generated via a process called back-splicing, where the 5′ splice donor site and 3′ splice acceptor site of the precursor mRNAs (pre-mRNAs) are covalently linked in a reverse order [12]. CircRNAs can be sorted into three main groups according to their different splicing products and processes: exonic circRNAs (EcRNAs), exon–intron circRNAs (EIciRNAs) and circular intronic RNAs (ciRNAs). The additional subgroups, intergenic circRNAs and tRNA intronic circRNAs, are small subgroups that are rarely studied [13] (Fig. 1).

During the mRNA transcriptional process, pre-mRNAs are spliced, introns are removed, and exons are connected alternatively. In addition, circRNAs are also generated from pre-mRNAs through different mechanisms, including lariat-driven circularization and intron-pairing-driven circularization [7, 14]. In the lariat-driven circularization model, lariat precursors are generated during exon skipping when final mRNA products are spliced out from alternative exons. EcRNAs are then formed after the intron sequence is removed by splicing within the lariat structure [15]. If the intron between exons is retained, the cyclizing transcript tends to form an ElciRNA. Under some circumstances, intron lariats that escape the process of intron debranching and degradation can lead to the formation of ciRNAs [16]. In the second circularization model, circularization is mediated by base pairing between reverse complementary sequences located in the flanking introns bordering the circularized exons. Alu elements are one of the repetitive complementary repeats and are highly abundant, existing in more than 10% of the human genome [17]. Compared to those with other origins, Alu elements derived from flanking introns are more likely to constitute a complementary site, which may bring the splice donor close to the acceptor and facilitate nucleophilic attack and cleavage [18].

Additionally, some RNA binding proteins (RBPs) might act as regulatory activators or inhibitors in circRNA biogenesis by interacting with specific binding sites in flanking intronic sequences of pre-mRNAs. For example, quaking (QKI) is an alternative splicing factor that is well known for its upregulation during epithelial-mesenchymal transition (EMT). It has been reported that QKI promotes the formation of circRNAs through intronic QKI binding motifs and dynamically modulates the production of more than one-third of circRNAs [19]. Another RBP, muscleblind (MBL), can interact with its own pre-mRNA and stimulate its circularization, leading to an increase in the ratio of circMBL to linear MBL [20]. In contrast, some RBPs act as negative regulatory factors. Adenosine deaminase acting on RNA (ADAR) is a double-stranded RNA-specific RNA-editing enzyme that can bind to Alu repeats in flanking introns. ADAR diminishes the complementarity and stability of these intron pair interactions through adenosine-to-inosine (A-to-I) editing activity, thus antagonizing circRNA biogenesis [18, 21]. Moreover, the nuclear RNA helicase Dexh-box helicase 9 (DHX9) can unwind RNA pairs flanking circularized exons, downregulating circRNA production [22].

In brief, multiple pathways and regulatory factors are involved in the biogenesis of circRNAs, and the relevant mechanisms are still unclear; thus, more research is needed to dig deeper into these processes.

## Functions of circRNAs

Emerging evidence suggests that circRNAs participate in different physiological and pathological processes at the transcriptional or posttranscriptional level [23, 24]. Here, we summarize the main functions of circRNAs (Fig. 1).

### CircRNAs act as post-transcriptional modulators

ElciRNAs and ciRNAs that regulate transcription are related to nuclear insoluble fractionation and tend to exist predominantly in the nucleus and contain few microRNA (miRNA) binding sites, thus differing from circRNAs, which are mainly located in the cytoplasm. Recent research has demonstrated that many of them affect alternative splicing by interacting with RNA polymerase II (Pol II), thereby managing the expression of parental genes. In particular, circANKRD52 silencing leads to a significant reduction in the transcription rate of ANKRD52 by associating with the elongation RNA Pol II complex [16]. Another study discovered that, when localized in the nucleus, circ-EIF3J and circ-PAIP2, identified as EIciRNAs, interacted with U1 small nuclear ribonucleic proteins (snRNPs) and further combined with the Pol II transcription complex at the promoters of parental genes to facilitate their expression. Conversely, a circRNA from the SEPALLATA3 gene was found to affect the splicing of its homologous mRNA through R-loop formation, causing a reduction in gene transcription [25]. In addition, more extensive research in Drosophila and human cells has shown that when linear splicing is increased, circRNA production decreases accordingly, indicating that there is a tendency to achieve endogenous equilibrium via the cyclization and linear splicing of pre-mRNAs [20, 26].

### CircRNAs undergo translational process of proteins

Most circRNAs consisting of exons are mainly present in the cytoplasm, giving them the ability to associate with ribosomes for translation into proteins [27]. Recently, strong evidence from many research groups has shown that a protein-coding circRNA should contain one or several of the following features: (a) an open reading frame (ORF) of sufficient length or the ability to undergo rolling circle translation; (b) an ORF spanning the back-splicing junction (BSJ), different from the linear transcript; and (c) necessary regulation elements for translation initiation upstream of the ORF, such as the internal ribosome entry site (IRES) element or N6-methyladenosine (m6A) modifications [28]. CircZNF609 is an example that has been reported in murine and human myoblasts. It contains a start-to-stop codon reading frame generated through a BSJ that is not present on related linear RNA molecules. Thus, it can be translated into a protein driven by IRES and identified by heavy polysomes in a splicing-dependent and cap-independent manner. Additionally, approximately 13% of circRNAs carry the m6A motif, which promotes the intracellular initiation of polypeptide translation from circRNAs. Moreover, the m6A reader protein yt521-b homology domain-containing family 3 (YTHDF3) recognizes the modification site of circRNAs and recruits eukaryotic translation initiation factor 4 gamma 2 (EIF4G2) and other translation initiation factors to drive the translation of circRNAs [29].

### CircRNAs function as competing endogenous RNAs (ceRNAs)

CeRNAs constitute a complex posttranscriptional regulatory network centered on miRNAs [30]. Apart from competing mRNAs, lncRNAs and transcribed pseudogenes, mounting evidence has confirmed that many circRNAs regulate the miRNA-mRNA network as ceRNAs [31]. Mechanistically, most identified circRNAs, such as EcRNAs, exist in the cytoplasm and colocalize with miRNAs. Indeed, circRNAs containing miRNA response elements (MREs) may bind miRNAs to reduce their activity, subsequently removing their inhibitory effect on the target mRNAs. The most extensively studied circular RNA, ciRs-7 (also termed CDR1), acts as a designated miR-7 inhibitor and was used to establish a conceptual mechanistic understanding of miRNA networks [32]. As a molecular sponge of miR-7, ciRs-7 harbors more than 60 conserved binding sites for miR-7, resulting in decreased miR-7 function and upregulation of miR-7 target genes. In situ profiling showed that miR-7 and ciRs-7 shared specific expression domains, indicating that miR-7 expressed in the brain is recruited by ciRs-7 [33]. Recently, a ceRNA sponging regulatory network involving the long noncoding RNA Cyrano, a ciRs-7 circRNA, and two miRNAs, miR-671 and miR-7, was reported, and this network suggests a new mechanism by which the crosstalk of multiple noncoding RNAs (ncRNAs) can regulate miRNAs [34]. In summary, many findings support and have contributed to the idea that circRNAs can function as ceRNAs and may be a universal biological phenomenon.

### CircRNAs bind to proteins

Some circRNAs contain conserved protein-binding sequences, which can be demonstrated by the colocalization of circRNAs and proteins. The interactions between them can be used to categorize their roles as protein decoys, scaffolds and recruiters; these interactions regulate the transcription of parental genes, facilitate the interaction of multiple proteins, and alter the subcellular localization of proteins. circRNAs can have one-to-one or one-to-many relationships with targets, forming different binding complexes under different circumstances [35]. CircFOXO3 might be the best example: it is downregulated in cancer cells and related to apoptosis and cell proliferation. It binds to cyclin-dependent kinase inhibitor 1 (p21) and cyclin-dependent kinase 2 (CDK2) and induces cell cycle arrest via the formation of a ternary complex. The interaction between p21 and CDK2 can be strengthened by circFOXO3, leading to the inhibition of CDK2 activity at the G1 and S phases [36]. In addition, several studies have found that circFOXO3 can also interact with the senescence-related proteins inhibitor of differentiation 1 (ID1) and e2f transcription factor 1 (E2F1) and the tumor-related proteins hypoxia inducible factor 1 alpha (HIF1α) and focal adhesion kinase (FAK). Moreover, in breast cancer, circFOXO3 can bind both p53 and mouse double minute 2 (MDM2), resulting in the occupation of MDM2 and enhanced p53 ubiquitination [37].

## Research approaches for circRNAs

Due to the potentially significant roles of circRNAs in disease diagnosis and prognosis, researchers are devoting more energy to investigating the genome-wide expression patterns of circRNAs. To date, circRNA microarrays and RNA sequencing are the two main techniques employed for genome-wide annotation of circRNAs.

Microarray analysis is a high-throughput technique that employs probes to identify specific circRNA junction sequences and quantify their expression. The advantage of this approach is the precise identification and quantification of specific circRNAs. However, circRNAs not within the target dataset could be missed. To date, these platforms have been developed with over 10,000 circRNA targets; for example, the Arraystar Human circRNA Array has been utilized in investigating multiple malignancies [38].

RNA sequencing is currently the most widely used method in circRNA research. Sequencing technology, which is different from microarrays, allows for the discovery of novel circRNAs that have not been previously identified by cloning or sequencing and reveals the actual structure of circRNAs [39]. However, due to their circular nature and extensive sequence overlapping with cognate linear transcripts, there are some specific challenges in detection and quantification of circRNAs, ranging from the initial RNA library establishment to the high algorithmic sensitivity required with low read counts in computational workflows.

The first challenge is the relatively low abundance of endogenous circRNAs compared to their linear counterparts [14]. CircRNAs lack polyadenylated (poly(A)) tails and possess a nonlinear conformation, which is abolished after the poly(A) + enrichment step in RNA-seq profiling. Currently, the widely used Ribo-Zero approach (which facilitates ribosomal RNA (rRNA) depletion) in sequencing library construction led to the discovery of thousands of circRNAs [40]. Furthermore, circRNAs can be enriched by the additional application of the 3′-5′ exonuclease Ribonuclease R (RNase R), which degrades linear RNA [41]. However, certain circRNAs are sensitive to RNase R, including CDR1as, MAN1A2 and NCX1, while some linear transcripts were found to be resistant to RNase R, such as small nuclear RNAs (snRNAs) and histone mRNAs [14, 42]. This biochemical variability could lead to inaccurate estimates of the genome-wide false-positive rate. Thus, there is great need to further develop a high-efficiency pretreatment assay for circRNA enrichment.

Another problem is the difficult trade-off between linear and circular RNAs. The Ribo-Zero library contains both poly(A) (linear) and nonpoly(A) (circular) RNAs after rRNA depletion, providing RNA information from a wider perspective to facilitate downstream or correlation analyses; however, it does not involve the tailored enrichment of circRNAs, which may increase the false positive rate. In contrast, RNase R digests linear RNA, while the covalently closed loop structure of circRNAs allows them to escape exonucleolytic degradation, resulting in the enrichment of circRNAs but the loss of some global information. Recently, exome capture RNA-seq was performed to detect circRNAs [43]. By targeting gene bodies, this strategy complemented conventional Ribo-Zero or RNase R strategies, circRNAs were highly enriched, and linear RNAs were simultaneously preserved. Although the resulting circRNAs were limited to known exonic regions, excluding circRNAs generated from intronic and intergenic regions, this method identified read-through circRNAs, a novel class of circRNAs involving exons that originate from multiple genes.

Following library preparation, bioinformatical challenges arise when dealing with the burst of RNA-seq data, specifically, the identification and differentiation of circRNAs from other RNA molecules. A variety of computational algorithms based on BSJs have recently been developed. These approaches can be categorized as split alignment-based or pseudoreference-based approaches [44]. The first tool category includes CIRCexplorer, CIRI, and find_circ, which split the reads spanning BSJs into segments and then align them to a reference sequence. The other category involves tools for constructing a pseudoreference based on all possible BSJs; the reads are aligned to this pseudoreference using algorithms such as KNIFE, NCLscan, and PTESFinder. However, an apparent BSJ could be generated from other cellular mechanisms, such as tandem DNA duplication or reverse transcriptase template switching. Moreover, one back-splicing sequence may represent diverse circRNAs containing different internal structures from a single parental gene. Considering the internal components of circRNAs, a recent new strategy, called reverse overlap (RO), has been proposed. The renewed CIRI-full algorithm combines both RO and BSJ reads, offering us a fresh perspective into circRNAs at the isoform level [45].

## Roles of circRNAs in PC

**Fig. 2:**
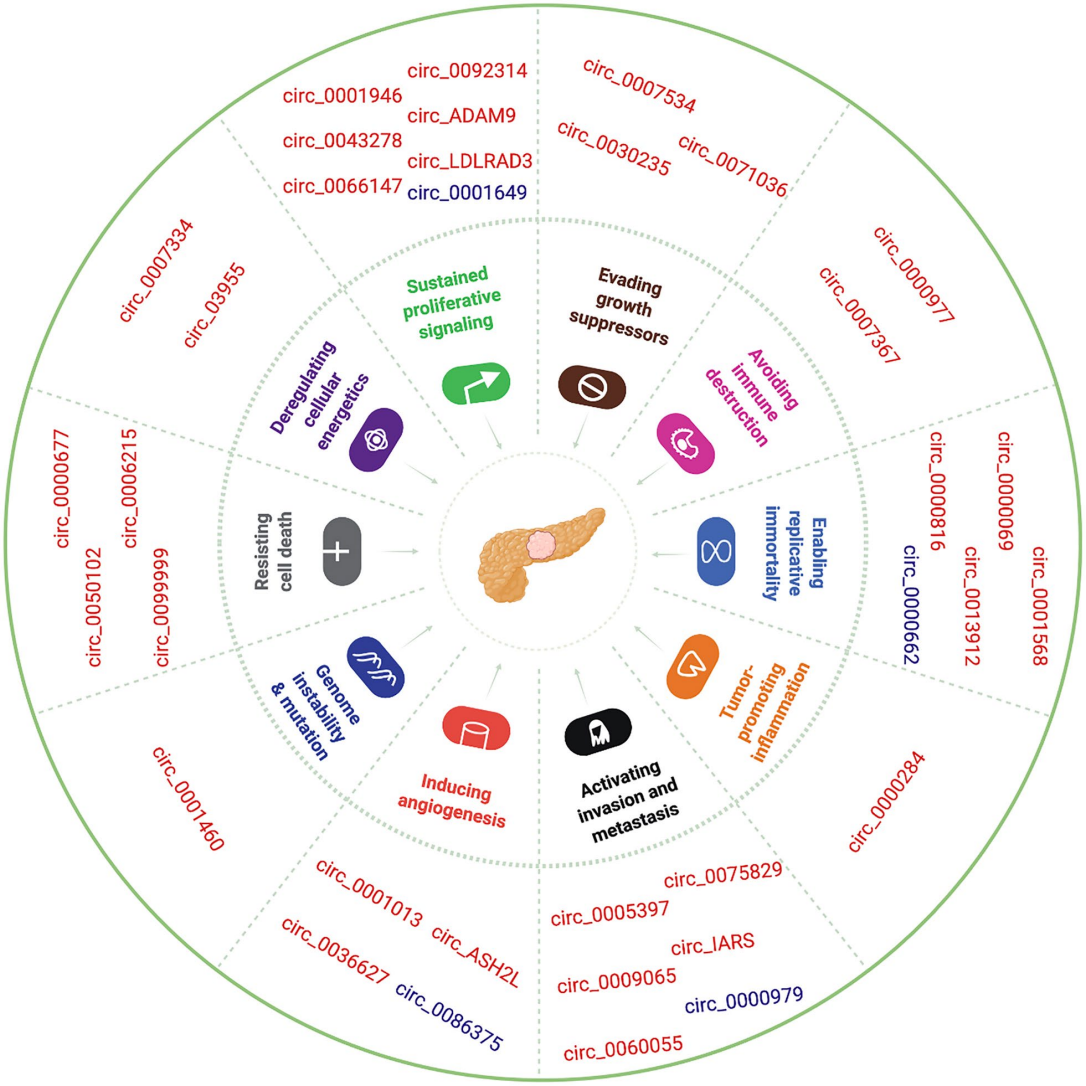
Functional role of PC-related circRNAs. CircRNAs are associated with the hallmarks of PC. The red markers indicate upregulated circRNAs, while the purple markers indicate downregulated circRNAs

Due to their functional diversity and the improvements in research methods, circRNAs have been increasingly revealed to play important roles in many human diseases, including PC. Therefore, this review summarizes the published circRNAs that are related to PC occurrence and development (Fig. 2).

## CircRNA profiles in PC

In recent years, with the development of high-throughput sequencing technology, circRNAs associated with PC have been gradually discovered (Table 1). Moreover, through bioinformatics exploration and laboratory verification, more potentially valuable circRNAs have been mined. In terms of source, PC tissues were used in 8 (8/16, 50.0%) of the published sequencing results. For example, Li et al. identified 5,396 differentially expressed circRNAs between 6 paired PC and adjacent nontumor tissues using a microarray [46]. Among them, 351 circRNAs were significantly differentially expressed in PC tissues compared with nontumor tissues (209 upregulated and 142 downregulated). The results have been uploaded to the public database under the number GSE69362. Guo et al*.* analyzed 20 paired of PC and adjacent nontumor tissues and identified 289 differentially expressed circRNAs (128 upregulated and 161 downregulated) with a fold change ≥ 2.0 and *P* < 0.05 as the threshold, and the final data were reported under the number GSE79634 [38]. Similarly, Han et al. and Xiong et al. also identified differentially expressed circRNAs in PC and adjacent nontumor tissues [47, 48]. Yang et al*.* identified 28,347 differentially expressed circRNAs using RNA sequencing in 5 pairs of PC and adjacent nontumor tissues, of which 278 were significantly differentially expressed circRNAs (173 upregulated and 105 downregulated) [49]. Kong et al. identified 13 significantly differentially downregulated circRNAs with a fold change < 0.4 and *P* < 0.05 as thresholds [50]. Similarly, Seimiya et al. and Shen et al. also identified a number of differentially expressed circRNAs [51, 52]. Among the datasets generated from these studies, GSE69362 and GSE79634 are the most widely used. This also suggests that in-depth mining of bioinformatics data, such as the exploration of overlapping circRNAs between these studies, will play a crucial role in our full understanding of PC-related circRNAs [38, 46]. Interestingly, Li et al. used exosomes from the plasma of PC patients or healthy people to analyze differentially expressed circRNAs [53]. A total of 453 differentially expressed circRNAs were identified and found to be associated with key cell biological processes and signaling pathways using gene ontology (GO) and Kyoto Encyclopedia of Genes and Genomes (KEGG) pathway analyses.

**Table 1:** Overview of circRNAs identified by microarrays and RNA sequencing in pancreatic cancer

No	Sample	Detection	Treatment	GEO	Total circRNAs	Cut-off	CircRNA differently expressed	Reference
1	6 PC tissues and 6 paired adjacent nontumor tissues	Microarray		GSE69362	5396	Fold change ≥ 1.5 and p < 0.05	351 (209 upregulated, 142 downregulated)	27,997,903
2	20 PC tissues and 20 paired adjacent nontumor tissues	Microarray	Rnase R	GSE79634		Fold change ≥ 2.0 and p < 0.05	289 (128 upregulated, 161 downregulated)	29,620,241
3	4 PC tissues and 4 normal pancreatic tissues	Microarray	Rnase R		11,471	Fold change > 1.5 and p < 0.05	193 (120 upregulated, 73 downregulated)	33,507,122
4	5 PC tissues and 5 normal pancreatic tissues	Microarray				Fold change > 2 and p < 0.05		33,593,338
5	5 PC tissues and 5 paired adjacent nontumor tissues	RNA sequencing	rRNA-depleted and Rnase R		28,374	Fold change ≥ 2.0 and p < 0.05	278 (173 upregulated, 105 downregulated)	31,428,151
6	5 PC tissues and 5 paired adjacent nontumor tissues	RNA sequencing		GSE136569		Fold change < 0.4 and p < 0.05	13 downregulated	32,366,257
7	2 PC tissues and 2 normal pancreatic tissues	RNA sequencing	Rnase R		58,050	false discovery rate < 0.05		32,879,441
8	3 PC tissues and 3 normal pancreatic tissues	RNA sequencing	Rnase R	PRJNA695439		Fold change > 1 and p < 0.05	203 (79 upregulated, 124 downregulated)	33,750,389
9	Exosomes of 8 PC plasma and 8 healthy volunteers’ plasma	RNA sequencing	rRNA-depleted and Rnase R			Fold change ≥ 2.0 and p ≤ 0.05	453 (274 upregulated, 179 downregulated)	31,605,569
10	SW1990 and SW1990-GEM resistant	Microarray	Rnase R			Fold change ≥ 2.0 and p < 0.05	81 (26 upregulated, 55 downregulated)	29,781,033
11	PANC1 and PANC1-GEM resistant	RNA sequencing		GSE1105801		Fold change ≥ 2.0 and p < 0.05	126 (68 upregulated, 58 downregulated)	29,922,161
12	PC cells with or without Ten Gy of X-ray radiation	RNA sequencing	Ribo-Zero rRNA Removal Kits		12,572	Fold change > 2 and p < 0.05	196 (182 upregulated, 14 downregulated)	32,727,565
13	PANC1 and PANC1-autophagic inhibition	Microarray			9420			30,570,107
14	PANC1 and PANC1-nigericin; SW1990 and SW1990-nigericin	RNA sequencing	remove ribosomal RNA	PRJNA543685		Fold change ≥ 2.0 and p < 0.05	183 (141 upregulated, 42 downregulated)	31,533,620
15	Exosomes of Hs766T and Hs766T-L2 cells	Microarray	RNase R					29,709,702
16	Stellate cells from 5 PC and 5 normal tissues	RNA sequencing				Fold change ≥ 2.0 and p ≤ 0.05	841 (388 upregulated, 453 downregulated)	33,042,405

PC: Pancreatic cancer; GEO: Gene expression omnibus

In addition to the direct use of clinical specimens, the sequencing of circRNAs related to PC cells is also increasing, which is of great importance in the exploration of the specific molecular mechanisms underlying PC development. Xu et al. and Shao et al. compared GEM-resistant PC cell lines with corresponding parental lines and found 81 and 126 circRNAs with significant differences in expression [54, 55]. Chen et al. investigated the differentially expressed circRNAs in PC cells with/without radiation therapy [56]. With autophagy inhibition or nigericin treatment, many significantly differentially expressed circRNAs were found [57, 58]. Differentially expressed circRNAs in PC cells derived from exosomes were also detected [59]. Finally, Shao et al. isolated stellate cells from 5 PC tissues and 5 normal pancreatic tissues and found a total of 841 differentially expressed circRNAs (388 upregulated and 453 downregulated) [60].

## Biological roles and molecular mechanisms of circRNAs in PC

Where do tumor cells come from? Why do tumor cells live forever? These two questions have always been the most popular and the most difficult in cancer research. With increasing research on circRNAs, we have found that some circRNAs play key regulatory roles in PC. Therefore, we have summarized the biological roles (Table 2) and molecular mechanisms (Table 3) of the circRNAs known to be associated with PC.

**Table 2:** Overview of cellular functions of circRNAs in pancreatic cancer

No	Circ	Host gene	Vitro Functions							Specimen	Expression	Reference
			Prolife	Cycle	Apopt	Migra	Invas	Angio	Other			
1	ciRS7	CDR1as	+				+			BXPC3/PANC1	Up	30,898,507
2	0000284	HIPK3	+							BXPC3	Up	29,255,366
3	0005273	PTK2	+			+				ASPC1/CFPAC1	Up	33,275,224
4	chr12:7,467,880,474,700,449		+							BXPC3	Up	32,879,441
5	0001649	SHPRH	+		+					BXPC3/PANC1	Down	29,969,694
6	ASH2L	ASH2L	+	+		+	+	+		CAPAN1/ASPC1	Up	31,718,694
7	0001460	NEIL3	+	+		+	+			CFPAC1/MIAPACA2	Up	33,750,389
8	0013912	POLR3C	+	+	+	+	+			ASPC1/PANC1	Up	32,884,344
9	0050102	PGPEP1	+	+	+	+	+			CFPAC1/PANC1	Up	33,289,016
10	0000662	AXIN1	+	+	+	+	+			ASPC1	Down	33,425,718
11	0000677	ABCC1	+		+	+	+			ASPC1/BXPC3	Up	33,413,045
12	0007534	DDX42	+		+	+				PANC1/SW1990	Up	30,382,592
13	0001946	CDR1as	+		+	+	+			ASPC1/PANC1	Up	33,593,338
14	0006215	SLC4A7	+		+	+				PANC1	Up	29,930,719
15	0060055	EIF6	+		+	+	+			HS766T/SW1990	Up	33,469,368
16	0066147	SFMBT1	+		+	+	+			BXPC3/PANC1	Up	32,855,541
17	0071036	INPP4B	+		+		+			ASPC1/PANC1	Up	33,507,122
18	0099999	ZMYM2	+		+		+			CFPAC1/PANC1	Up	30,537,731
19	IARS	IARS							Endothelial permeability	Exo-HUVEC/ASPC1/HS766T/HS766T-L2	Up	30,064,461
20	0036627	PDE8A	+			+	+	+	Scatteration	BXPC3/CAPAN2/Exo-HS766T	Up	29,709,702
21	ADAM9	ADAM9	+			+	+			CAPAN1/MIAPACA2	Up	31,810,373
22	0000069	STIL	+	+	+	+	+			MIAPACA2/SW1990; Exo-HPDE/SW1990	Up	33,324,055
23	0009065	BFAR	+			+	+			BXPC3/PANC1	Up	32,375,768
24	0043278	TADA2A	+			+	+			PANC1/SW1990	Up	33,505,218
25	0075829	CASC15	+			+	+			BXPC3/SW1990	Up	33,184,989
26	chr7:154,954,255,154,998,784		+			+	+			PANC1 + NAPSC/CAPSC	Up	33,042,405
27	LDLRAD3	LDLRAD3	+			+	+			PANC1/SW1990	Up	31,521,692
28	0001013	KIAA1841	+			+	+	+		ASPC1/PANC1	Up	33,563,550
29	0001568	DUSP22	+	+	+		+	+		BXPC3/CAPAN2	Up	32,193,152
30	0000979	TCONS_00003590	+			+	+	+		PANC1	Down	32,878,470
31	0086375	NFIB				+		+		CAPAN2/PANC1	Down	32,366,257
32	circ 03955		+		+				Glycolysis	BXPC3/PANC1	Up	33,864,618
33	0007334	MBOAT2	+		+	+	+		Glutamine metabolism	PANC1/SW1990	Up	33,832,516
34	0000977	NOL10							Inhibition of NK cells under hypoxia	PANC1/NK cell	Induced by hypoxiain	31,402,756
35	0007367	UBAP2							Immune infiltration	GSE69362 and GSE79634	Up	31,584,877
36	0002130	C3								PANC1/SW1990; PDX tumor	Up	32,727,565
37	0092314	RANBP1	+				+		CSC	ASPC1/PACA2	Up	33,842,379
38	0005397	RHOT1	+			+	+			CAPAN2/PANC1	Up	30,444,423
39	0007334	MBOAT2				+				PANC1		31,428,151
40	0030235	RCBTB2	+		+	+	+			PANC1/SW1990	Up	30,591,218
41	0000816	FOXK2	+	+	+	+	+			CFPAC1/PANC1	Up	32,217,695
42	0006988	LDLRAD3								ASPC1/CAPAN2/HPCY5/HPDE6C7/PANC1/SW1990	Up	29,307,994
43	chr14:101,402,109,101,464,448	SNHG23			+				GEM-resistance	PANC1-GEM	Up	29,922,161
44	chr4:5,272,960,352,780,244	DCUN1D4			+				GEM-resistance	PANC1-GEM	Up	29,922,161
45	0000284	HIPK3	+		+	+	+		GEM-resistance	PANC1/SW1990-GEM	Up	32,104,074
46	101672/004077/003251								GEM-resistance	SW1990-GEM	Up	29,781,033
47	101543/002747/000926								GEM-resistance	SW1990-GEM	Down	29,781,033

Prolife: Proliferation; Cycle: Cell cycle; Apopt: Apoptosis; Migra: Migration; Invas: Invasion; Angio: Angiogenesis; CSC: Cancer Stem Cell; GEM: Gemcitabine

**Table 3:** Overview of mechanisms and animal studies of circRNAs in pancreatic cancer

No	Circ	Position	Location	Mechanisms				Vivo functions		Pheno	Reference
				miRNAs	Targets	RBPs	Pathways	Grow	Meta		
1	ciRS-7	chrX:q27.1		miR-7	EGFR		STAT3			Onco	30,898,507
2	0000284	chr11:33,307,958–33,309,057		miR-124	IL-6		JAK-STAT3	+		Onco	29,255,366
3	0005273	chr8:141,710,989–141,716,304				KLF12				Onco	33,275,224
4	chr12:74,678,804–74,700,449	chr12:74,678,804–74,700,449	Cyto	none						Onco	32,879,441
5	0001649	chr6:146,209,155–146,216,113								Suppr	29,969,694
6	ASH2L		Cyto (mainly) and nucl	miR-34a	Notch1		EMT; VEGF	+	+	Onco	31,718,694
7	0001460	chr4:178,274,461–178,281,831	Cyto	miR-432-5p	ADAR1		GLI1 (A to I RNA-editing)-EMT/CCND; negative regulation of circNEIL3	+	+	Onco	33,750,389
8	0013912	chr1:145,601,529–145,601,852	Cyto	miR-7-5p			EMT	+		Onco	32,884,344
9	0050102	chr19:18,459,757–18,466,821	Cyto	miR-1182	NPSR1			+		onco	33,289,016
10	0000662	chr16:398,402–398,484	Cyto	miR-361-3p	BTG2			+		Suppr	33,425,718
11	0000677	chr16:16,101,672–16,162,159								Onco	33,413,045
12	0007534	chr17:61,869,771–61,877,977		miR‐625/892b			Bcl2/Bax; MMP2	+		Onco	30,382,592
13	0001946	chrX:q27.1		miR-432-5p	E2F3			+		Onco	33,593,338
14	0006215	chr3:27,478,878–27,490,288		miR-378-3p	SERPINA4					Onco	29,930,719
15	0060055	chr20:33,866,724–33,872,064		miR-557	SLC7A11		PI3K/AKT	+		Onco	33,469,368
16	0066147	chr3:52,960,046–52,962,357	Cyto	miR-330-5p	PAK1		EMT	+	+	Onco	32,855,541
17	0071036	chr4:143,324,090–143,326,477	cyto (mainly) and nucl	miR-489				+		Onco	33,507,122
18	0099999	chr13:20,633,586–20,638,685		miR-335-5p	JMJD2C			+		Onco	30,537,731
19	IARS			miR-122	RhOA		ZO1; F-actin	+	+	Onco	30,064,461
20	0036627	chr15:85,656,607–85,669,605	Cyto (mainly) and nucl	miR-338	MACC1		MET	+	+	Onco	29,709,702
21	ADAM9			miR-217	PRSS3		ERK/VEGF	+		Onco	31,810,373
22	0000069	chr1:47,745,912–47,748,131	Cyto	miR-144	STIL			+		Onco	33,324,055
23	0009065	chr16:14,738,130–14,738,466	Cyto	miR-34b-5p	MET		AKT	+	+	Onco	32,375,768
24	0043278	chr17:35,797,838–35,800,763	Cyto	miR-455-3p	CD80		cytokines	+		Onco	33,505,218
25	0075829	chr6:22,020,567–22,056,919	Cyto	miR-1287-5p	LAMTOR3		ERK/AKT	+	+	Onco	33,184,989
26	chr7:154,954,255–154,998,784	chr7:154,954,255–154,998,784		miR-4459			KIAA0513	+		Onco	33,042,405
27	LDLRAD3			miR-137-3p			PTN	+		Onco	31,521,692
28	0001013	chr2:61,339,656–61,345,251	Cyto	miR-145	KLF5		MMP; VEGF	+		Onco	33,563,550
29	0001568	chr6:349,113–349,256	Cyto (mainly) and nucl	miR-377	HOXC6		PCNA; VEGF; MMP; caspase3	+		Onco	32,193,152
30	0000979	chr2:19,042,277–19,042,456	Cyto	miR-223	SLC4A4		EMT; MMP; VEGF	+		Suppr	32,878,470
31	0086375	chr9:14,146,687–14,155,892	Cyto	miR-486-5p	PIK3R1		PI3K/VEGF	+	+	Suppr	32,366,257
32	03955			miR-3662	HIF-1a			+		Onco	33,864,618
33	0007334	chr2:9,083,315–9,098,771	Cyto	miR-433-3p	GOT1			+		Onco	33,832,516
34	0000977	chr2:10,784,445–10,808,849		miR-153	HIF-1a; ADAM10					Onco	31,402,756
35	0007367	chr9:33,948,371–33,956,144		miR-494			CXCR4/ZEB1			Onco	31,584,877
36	0002130	chr19:6,702,137–6,702,590		miR-4482-3p	NBN					Onco	32,727,565
37	0092314	chr22:20,113,099–20,113,439	Cyto	miR-761	S100P		AKT/EMT	+		Onco	33,842,379
38	0005397	chr17:30,500,849–30,503,232	Cyto	miR-26b-3p/125a-3p/330-5p/382-5p			MAPK; Wnt; Ras			Onco	30,444,423
39	0007334	chr2:9,083,315–9,098,771		miR-144-3p/miR-577	MMP7; COL1A1					Onco	31,428,151
40	0030235	chr13:49,075,877–49,077,050		miR-1253/miR-1294						Onco	30,591,218
41	0000816	chr17:80,521,229–80,526,077	Cyto	miR-942	ANK1; GDNF; PAX6	YBX1; hnRNPK	NUF2; PDXK	+	+	Onco	32,217,695
42	0006988	chr11:36,248,634–36,248,980								Onco	29,307,994
43	chr14:101,402,109–101,464,448	chr14:101,402,109–101,464,448		miR-145-5p						Onco	29,922,161
44	chr4:52,729,603–52,780,244	chr4:52,729,603–52,780,244		miR-145-5p						Onco	29,922,161
45	0000284	chr11:33,307,958–33,309,057		miR-330-5p	RASSF1		EMT	+		Onco	32,104,074
46	101672/004077/003251									Onco	29,781,033
47	101543/002747/000926									Suppr	29,781,033

cyto: cytoplasm; nucl: nucleus; miRNAs: microRNAs; RBPs: RNA binding proteins; Meta: Metastasis; Pheno: Phenomenon; onco: oncogene; suppr: suppressor

### CircRNAs affect the proliferation of PC

**Fig. 3:**
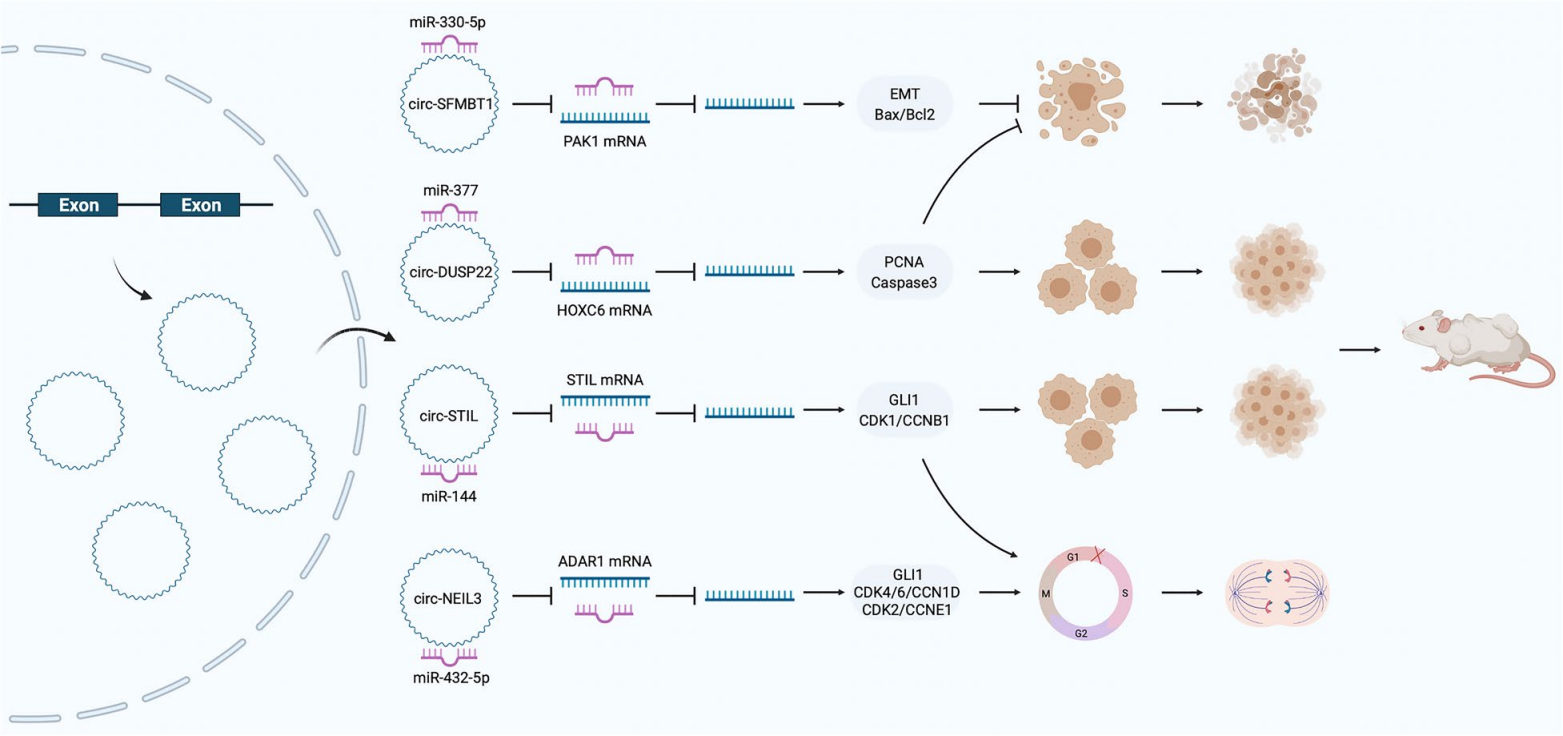
The specific mechanism of circRNAs in the proliferation, cell cycle, and apoptosis of PC cells

The most basic biological characteristic of tumor cells is their unlimited ability to proliferate (Fig. 3). Liu et al. found that ciRs-7 was highly expressed in PC tissues and cells. Bioinformatics predicted and confirmed that ciRs-7 could adsorb miR-7 to promote the expression of epidermal growth factor receptor (EGFR) and promote the proliferation of PC cells [61]. Chen et al. demonstrated that hsa_circ_0000284 promotes IL-6 expression through adsorption of miR-124 in BXPX3 cells and then promotes cell proliferation by upregulating the JAK/STAT3 signaling pathway. These results were also confirmed in nude mice [62]. Recent studies have shown that the IL-6/JAK/STAT3 signaling pathway can affect the prognosis of non-small-cell lung cancer and glioma by regulating immunosuppression. Hou et al*.* found that hsa_circ_0005273 was highly expressed in PC tissues and cells. Specifically, hsa_circ_0005273 was found to promote the proliferation of ASPC1 and CFPAC1 cells by negatively regulating the RNA-binding protein KLF12 which could also restore the effect of this circRNA on PC cells [63]. Seimiya et al. found that circ_chr12:74,678,804–74,700,449 was highly expressed in PC tissues and demonstrated that it could promote proliferation in BXPC-3 cells. In contrast, Jiang et al. found that hsa_circ_0001649 is expressed at low levels in PC tissues and inhibits proliferation and promotes apoptosis in BXPC-3 and PANC1 cells [51, 64]. However, the specific mechanism by which they play roles in regulating PC cells proliferation remains unclear and needs further research.

The cell cycle refers to the normal process of cell replication and reproduction and includes the prophase of DNA synthesis (G1 phase), DNA synthesis phase (S phase), DNA synthesis phase (G2 phase), and mitotic phase (M phase). The entire process is controlled and regulated by cyclins and cyclin-dependent kinases [65]. Increasing evidence has shown that the occurrence and development of PC is closely related to disruption of the cell cycle [66]. Chen et al. analyzed the differential expression of circRNAs in PC cells and their derived exosomes and found that circ-ASH2L was enriched only in PC cells [67]. Further studies showed that this circRNA was significantly increased in PC tissues and cells and was mainly localized in the cytoplasm. After forced upregulation of circASH2L, the oncogenic ability of PC cells was significantly enhanced, including an increase in the proportion of G1-phase cells, and a decrease in the proportion of G2-phase cells. In vivo experiments showed that circ-ASH2L also promoted tumor formation and distant metastasis in nude mice. Mechanistically, circ-ASH2L activates the Notch1 signaling pathway through the adsorption of miR-34a, thereby promoting PC development. Shen et al. found that hsa_circ_0001460 is a circRNA derived from exons 8 and 9 whose parent gene is NEIL3; its expression is increased in PC tissues and cells versus normal controls [52]. Functionally, knockdown of hsa_circ_0001460 inhibited the proliferation, migration, and invasion of PC cells and increased the proportion of G1-phase cells. Mechanistically, hsa_circ_0001460 acted as a sponge of miR-432-5p and promoted the expression of ADAR1. Interestingly, ADAR1 promotes the A-to-I conversion of glioma-associated oncogene 1 (GLI1) in exon 12 (chr12:57,864,624), thereby weakening the binding of the C-terminus to suppressor of fusion (SUFU) [68]. Eventually, the expression of the downstream targets (the cyclin D1/CDK4/CDK6 complex and cyclin E/CDK2 complex) was upregulated. The former complex inactivates protein kinase phosphorylation, limits the ability of cells to leave S phase, and promotes proliferation. The latter complex can cause S-phase cell cycle arrest and promote proliferation through the phosphorylation of downstream substrates. In addition, hsa_circ_0013912 and hsa_circ_0050102 were also confirmed to increase the proportion of G1-phase cells and promote the proliferation of PC cells [69, 70]. Huang et al. found that the low expression of hsa_circ_0000662 in PC tissues and cells plays a role in inhibiting the proliferation, migration and invasion of PC cells [71]. Moreover, overexpression of hsa_circ_0000662 significantly increased the proportion of G0/G1-phase cells but reduced the proportion of S-phase cells and promoted the apoptosis of AsPC-1 cells. Using fluorescence in situ hybridization (FISH), Huang et al. found that hsa_circ_0000662 was mainly located in the cytoplasm, further confirming that this circRNA can promote the expression of the tumor suppressor gene b-cell translocation gene 2 (BTG2) by adsorbing miR-361-3p.

Apoptosis is a natural cell death process under a normal physiological state, and the occurrence and development of tumor cells, including PC cells, are closely related to apoptosis dysregulation [72]. Hsa_circ_0000677, also known as hsa_circ_001569, has the parent gene ABCC1. Hsa_circ_0000677 is highly expressed in hepatocellular carcinoma and colorectal cancer and plays an adverse role [73, 74]. Shen et al. found that hsa_circ_0000677 is also highly expressed in PC tissues and cells [75]. After knockdown of hsa_circ_0000677 expression, the proliferation, migration and invasion ability of PC cells were significantly reduced, but the proportion of apoptotic cells was significantly increased. These results suggest that hsa_circ_0000677 might play a role in promoting carcinogenesis and development in PC cells. has_circ_0007534, located on chromosome 17, is a circRNA produced by exons 4 and 7 that is abnormally expressed in colorectal cancer and cervical cancer [76, 77]. Hao et al. found that this circRNA is also highly expressed in PC tissues and cells [78]. Bioinformatics predicted that this circRNA has binding sites for miR-625 and miR-892b, and luciferase reporter assays confirmed the ceRNA role of this circRNA. Functionally, knockdown of hsa_circ_0007534 promoted the apoptosis of PC cells, resulting in decreased expression of the antiapoptotic factor Bcl-2 and increased expression of the proapoptotic factor Bax. In addition, Xiong et al. found that hsa_circ_0001946 can adsorb miR-432-5p, and Zhu et al. found that hsa_circ_0006215 can adsorb miR-378-3p to inhibit the apoptosis of PC cells [48, 79]. The same is true for hsa_circ_0060055, hsa_circ_0066147, hsa_circ_0071036, and hsa_circ_0099999 [47, 80–82]. In contrast, Jiang et al. found that hsa_circ_0001649 can promote apoptosis by activating caspase-3 and caspase-9, thereby inhibiting the proliferation of PC cells [64]. The caspase family can specifically cleave the aspartic acid peptide bond; specifically, caspase-3 can be activated by caspase-9 through a variety of pathways, including mitochondrial pathways, to cleave a variety of structural and functional proteins in cells, thus leading to programmed cell death [83].

### CircRNAs affect the progression of PC

**Fig. 4:**
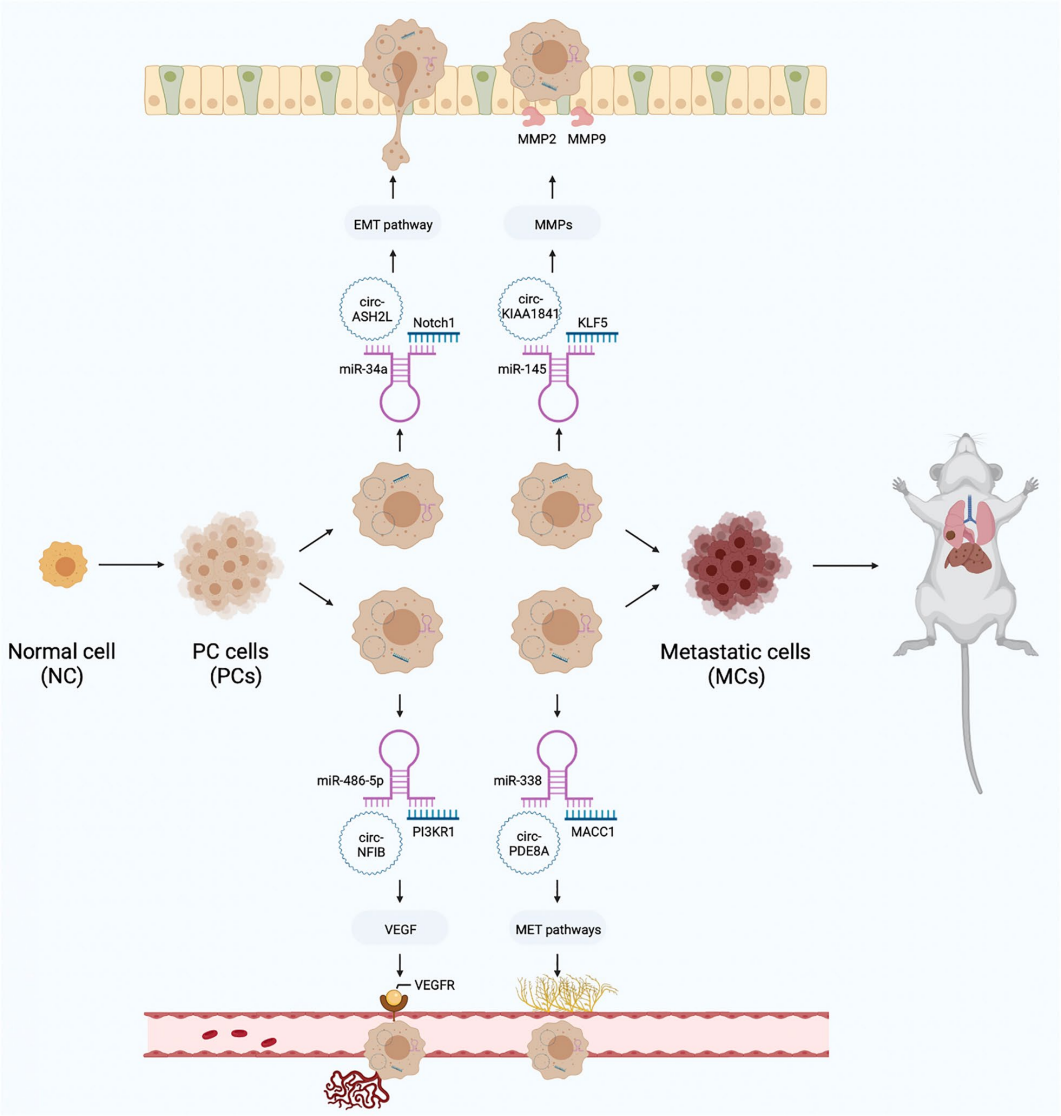
The specific mechanism of circRNAs in the migration, invasion, angiogenesis, and metastasis of PC cells

PC cells are rich in stroma and have strong invasion and metastasis abilities, which contribute to the high malignancy of PC [84]. Therefore, it is important to find molecular targets that can effectively inhibit PC progression to improve prognosis (Fig. 4). Li et al*.* found that circ-IARS was not only upregulated in PC tissues but also had higher expression levels in plasma exosomes from patients with distant metastasis, suggesting that this circRNA might be associated with PC metastasis [85]. According to Liotta, tumor invasion involves three steps: cell adhesion and deadhesion, extracellular matrix proteolysis and anti-proteolysis, and cell migration [86]. Furthermore, it has been confirmed that circ-IARS is enriched in signaling exosomes secreted by tumor cells and can be transferred to endothelial cells to act as a sponge to adsorb miR-122 and weaken its inhibition of ras homolog family member A (RhoA) [85]. Activated RhoA can activate F-actin to increase cell contraction and inhibit ZO1, leading to increased endothelial cell permeability and metastasis. When Li et al., using in vitro analysis, analyzed differentially expressed circRNAs in the exosomes of HS766T-L2 cells, second-generation primary tumor cells derived from liver metastases of HS766T cells, and HS766T cells (a human PC cell line), they found that hsa_circ_0036627 was highly expressed in PC tissues and plasma exosomes from patients [59]. Interestingly, this circRNA was found to promote tumorigenesis and liver metastasis in nude mice, and exosomes carrying this circRNA were found among red blood cells obtained via the tail vein. In terms of mechanism, bioinformatics and RNA-binding protein immunoprecipitation (RIP) assays confirmed that hsa_circ_0036627 and miR-338 bind to each other to upregulate the expression of metastasis associated in colon cancer 1 (MACC1), thereby activating the tyrosine kinase receptor MET. MET is one of the typical oncogenes in human epithelial cell carcinomas, including PC, and plays a procancer role by regulating the PI3K/AKT and RAS/MAPK signaling pathways through the SH2 domain [87]. Many other studies have also found that the expression of circRNAs is increased in PC cells, which can promote PC progression [60, 88, 89].

Tumor growth depends on the nutrition and support of tumor blood vessels [90]. Although PC tissues lack a blood supply, their microvascular density is significantly higher than that of normal tissue, which plays an important role in their development, especially metastasis [91]. Liu et al. found that the increased expression of hsa_circ_0001013 in PC tissues and cells could activated Kruppel-like factor 5 (KLF5) by downregulating miR-145 to promote PC cell proliferation, migration and angiogenesis [92]. Zhang et al. found that hsa_circ_0001568 can also promote angiogenesis in PC cells, and the specific mechanism may be related to circRN-mediated inhibition of human homeobox C6 (HOXC6) expression through binding of miR-377 [93]. Previous studies have shown that the mechanisms regulating angiogenesis include but are not limited to the following two mechanisms. The angiogenic factor VEGF, also known as vascular permeability factor, plays a central role in tumor angiogenesis by activating the VEGF/VEGFR axis [94]. In addition, the matrix metalloproteinase (MMP) family can degrade and reshape the extracellular matrix, which not only promotes tumor invasion but also plays an important role in angiogenesis [95]. Both hsa_circ_0001013 and hsa_circ_0001568 have been shown to upregulate the expression of VEGF, MMP2 and MMP9 to promote angiogenesis [92, 93]. In contrast, hsa_circ_0000979 and hsa_circ_0086375 were found to inhibit the expression of related pathways and thus block tumor progression [50, 96].

### CircRNAs affect other properties of PC

In 2011, Hanahan et al. proposed ten characteristics of tumor cells [97]. We found that circRNAs also play important roles in other areas of PC.

The function of normal cells is mainly mediated by the aerobic decomposition of glucose, and tumor cells have been found to rely on glycolysis to produce energy despite the presence of oxygen [98]. Liu et al*.* found that circ-03955 was highly expressed in PC tissues and cells and that this circRNA could bind to miR-3662 [99]. HIF1α was predicted to be a transcriptional target of miR-3662. HIF1α can upregulate pyruvate dehydrogenase kinase 1 and then inhibit pyruvate dehydrogenase, thereby inhibiting the tricarboxylic acid cycle, namely, aerobic decomposition. In addition, most of the key enzymes in glycolysis are positively regulated by HIF1α [100]. The results showed that under the adsorption of circRNA, the glycolysis level was significantly increased, and the apoptosis of PC cells was inhibited through HIF1α upregulation. Zhou et al. found that hsa_circ_0007334 plays a role in promoting glutamine metabolism through the miR-433-3p/GOT1 axis [101]. As an important carbon and nitrogen source in the process of cell growth, glutamine disorder can provide more abundant energy for tumor cells and promote their proliferation and metastasis [102]. Currently, research on circRNAs associated with energy metabolism in PC is still in its infancy, and further molecular mechanisms remain to be explored.

One of the reasons for the malignant progression of PC is that tumor cells can evade immune surveillance and avoid cell death or apoptosis. Studies have shown that high immune cell infiltration leads to immunosuppression throughout PC development [103]. Ou et al. found that hypoxia can induce hsa_circ_0000977 expression and then upregulate the expression of HIF1α and ADAM10 via the adsorption of miR-153 [104]. This led to a decrease in natural killer group 2 member d (NKG2D) binding to membrane-bound MHC class I polypeptide-related sequence a (mMICA), which inhibited the activation of effector NK cells and played a role in immune escape. In analysis of the GSE69362 and GSE79634 datasets, Zhao et al. found that hsa_circ_0007367 was highly expressed in PC [105]. Bioinformatics was used to screen CXCR4 and zinc finger E-box-binding protein 1 (ZEB1) as potential targets for hsa_circ_0007367. Further investigation showed that CXCR4 and ZEB1 were positively correlated with the majority of immune cells in PC cells, especially tumor-associated macrophages (TAMs), monocytes, and regulatory T cells; however, all the above results are theoretical and need further confirmation. Chemotherapy resistance is also associated with poorer outcomes in PC patients. We pooled our findings and found that a total of 5 PC-related circRNAs (shown in Table 2) were strongly associated with GEM resistance in 3 studies [54, 55, 106].

### Molecular mechanisms of circRNAs in PC

Most PC-associated circRNAs that have been reported are localized in the cytoplasm and act as ceRNAs. Hsa_circ_0002130 was reported to be upregulated in PC cell-derived exosomes irradiated at a dose of 10 Gy and was predicted to have binding sites for miR-4482-3p, and nibrin (NBN), which is associated with PC prognosis, was identified as a possible downstream target of miR-4482-3p [56]. Shen et al*.* found that hsa_circ_0092314 could adsorb miR-761 and promote S100P expression to regulate PC stem cell characteristics and promote invasion [107]. In addition, some circRNAs have multiple miR-binding sites; for example, hsa_circ_0005397 was predicted to bind with miR-26b-3p, miR-125a-3p, miR-330-5p, and miR-382-5p; hsa_circ_0007334 can bind to miR-144-3p and miR-577; and hsa_circ_0030235 can adsorb miR-1253 and miR-1294 to regulate PC formation and development [49, 108, 109]. On the other hand, a miRNA can be targeted by multiple circRNAs in PC; for example, miR-432-5p binds to both hsa_circ_0001460 and hsa_circ_0001946 [48, 52], and miR-330-5p can bind to both hsa_circ_0000284 and hsa_circ_0066147 [81, 106]. This nonunique binding mode provides circRNAs with greater cross reactivity and broader function.

In addition to their role as ceRNAs, in recent years, circRNAs have also been found to bind to RBPs and even encode proteins to exert effects in glioma, gastric cancer and colorectal cancer [110–112]. Wong et al*.* found that hsa_circ_0000816 can regulate the ANK1/GDNF/PAX6 pathway by adsorbing miR-942 and, using RNA pulldown preliminary screening, mass spectrometry analysis and RIP identification, confirmed that this circRNA can bind to the y-box binding protein 1 (YBX1)-heterogeneous nuclear ribonucleoprotein K (hnRNPK) complex [113]. Thus, hsa_circ_0000816 can upregulate the expression of the downstream target genes ndc80 kinetochore complex component (NUF2) and pyridoxal kinase (PDXK) to promote PC occurrence and development.

## Clinical significance of circRNAs in PC

PC has an insidious onset, lacks specific clinical manifestations, and has a 5-year survival rate of only 10% [1, 114]. Moreover, PC ranks first in mortality rate among digestive tumors in the United States. Due to the lack of effective methods for early diagnosis, more than 80% of patients diagnosed with PC have missed the opportunity for surgery by the time they are first diagnosed [115]. Currently, noninvasive enhanced computed tomography (CT) is recommended as the preferred diagnostic method, which can not only determine the size of the tumor but can also aid in the evaluation of resectability. Nevertheless, the sensitivity for tumors ≤ 2 cm is only 77%, and the diagnostic efficacy for patients with distant metastases is not as good as that of more expensive PET-CT imaging [116]. In humoral testing, CA19-9 is the only biomarker approved by the Food and Drug Administration (FDA) for diagnostic use [117]. However, CA19-9 is also elevated in other digestive system tumors and benign diseases, with a sensitivity of 70% to 92% and a specificity of 68% to 92% for PC [118]. Therefore, there is an urgent need for biomarkers for early PC diagnosis and early detection of postoperative recurrence and metastasis in PC.

In recent years, the beneficial role of body fluid biopsy, which provides samples, including circulating tumor DNA (ctDNA), circulating tumor cells (CTCs) and exosomes, for tumor diagnosis and treatment has been gradually revealed [119]. CircRNAs are highly valuable for the early diagnosis and prognosis evaluation of PC due to their tissue-specific and stage-specific properties. In addition, circRNAs are not easily destroyed by RNA hydrolases due to their circular structure and can stably exist in human blood and urine; thus, they can be used in new noninvasive diagnostic methods [120].

### Correlation between circRNAs and clinical variables of PC

In recent years, circRNAs have shown great potential in clinical application of PC [121]. Li et al. revealed that hsa_circ_0001946 and hsa_circ_0005397 were upregulated in the sequencing results, while hsa_circ_0006913, hsa_circ_0000257, hsa_circ_0005785, hsa_circ_0041150 and hsa_circ_0008719 were downregulated [46]. These results were validated in 20 pairs of PC tissues and paracancerous tissues. In addition, Guo et al*.* found that circ_100433 and 9 other circRNAs were upregulated and that circ_000167 was downregulated in PC tissues [38]. Sequencing results were subsequently confirmed by qRT-PCR analyses of 10 pairs of clinical specimens. In addition, other circRNAs, such as hsa_circ_0006215, hsa_circ_0066147, hsa_circ_0007334, and hsa_circ_0000816, have been shown to be highly expressed in PC tissues [49, 79, 81, 113]. CircRNA detection in blood is an emerging diagnostic method that is relatively noninvasive compared to tissue biopsy or resection [122]. In an analysis of differentially expressed circRNAs between plasma from PC patients and healthy controls (14 pairs of clinical specimens), Li et al. found and confirmed that six circRNAs, including hsa_circ_0002130, were highly expressed, and that four circRNAs, such as hsa_circ_0103896, were expressed at low levels in PC patients [53]. Liu et al. found increased expression of hsa_circ_0001013 in the plasma of PC patients [92]. In addition, using a preamplification method followed by qPCR, Seimiya et al. found that circ-chr12:74,678,804–74,700,449 was highly expressed in PC tissues and that the expression of this circRNA was related to lymph node metastasis and tumor-node-metastasis (TNM) stage [51]. Interestingly, this circRNA was positively expressed in the serum of PC patients and intraductal papillary mucinous neoplasm (IPMN) patients but was not expressed in healthy controls. These results suggest that circRNAs are differentially expressed in tissues or blood and have clinical guidance potential in PC patients.

Important clinical variables in PC include tumor size, differentiation grade, TNM stage, and vessel invasion. Liu et al*.* found that high ciRS-7 expression was associated with lymph node metastasis and venous invasion, suggesting that ciRS-7 can promote the clinical progression of PC [61]. Guo et al. found that hsa_circ_0013912, which is mainly located in the cytoplasm, was highly expressed in PC cells and might play a role in promoting cancer progression by regulating the EMT pathway through adsorption of miR-7-5p [69]. Hsa_circ_0013912 was shown to be more highly expressed in 54 PC tissues than in paracancerous tissues and was positively associated with lymph node metastasis and a poorer TNM stage. Yang et al. found that the expression of hsa_circ_0006988 increased successively in normal pancreatic cell lines (HPCY5 and HPDE6C7), PC cell lines (CAPAN2 and PANC1) and metastatic PC cell lines (ASPC1 and SW1990), suggesting that this circRNA may be related to PC occurrence and progression [123]. Subsequently, it was confirmed that this circRNA was highly expressed in 30 PC cases and in paracancerous tissues, and the expression level was related to lymphatic invasion and venous invasion. Moreover, Spearman analysis found that hsa_circ_0006988 was negatively correlated with T classification and clinical stage. Finally, this circRNA was found to be increased in the plasma of PC patients and negatively correlated with CA19-9 levels and distant metastasis. The area under the receiver operating characteristic (ROC) curve (AUC) of CA19-9 in plasma alone for PC diagnosis was 0.83 but was 0.87 after combination with hsa_circ_0006988 (sensitivity = 0.8033, specificity = 0.9355). The relationships between other circRNAs and clinical variables in PC are detailed in Tables 4, 5.

**Table 4:** Overview of prognostic, diagnostic and clinicopathological significance of circRNAs in pancreatic cancer tissues

No	Circ	Expression	Cut-off	Detect	Variables								AUC	Survival	Prognostic biomarker	Reference
					Size	Differentiation	T	Lymphatic (N)	Distance (M)	TNM	Vessel invasion	Other				
1	0001946/0005397	Up		qRT-PCR												27,997,903
2	0006913/0000257/0005785/0041150/0008719	Down		qRT-PCR												27,997,903
3	100433/101717/102049/102051/102619/103076/103390/104084/104270	Up		qRT-PCR												29,620,241
4	000167	Down		qRT-PCR												29,620,241
5	0001946	Up		qRT-PCR												33,593,338
6	0006215	Up		qRT-PCR												29,930,719
7	0066147	Up		qRT-PCR												32,855,541
8	0007334	Up		qRT-PCR												31,428,151
9	0000816	Up		qRT-PCR												32,217,695
10	chr12:74,678,804–74,700,449	Up		qRT-PCR and FISH				+		+						32,879,441
11	ciRS-7	Up		qRT-PCR				+			+					30,898,507
12	0013912	Up		qRT-PCR				+		+						32,884,344
13	0006988	Up		qRT-PCR			+	+		+	+					29,307,994
14	0050102	Up		qRT-PCR						+						33,289,016
15	0000662	Down		qRT-PCR						+						33,425,718
16	0099999			qRT-PCR				+								30,537,731
17	0075829	Up		qRT-PCR	+			+								33,184,989
18	0007334	Up		qRT-PCR						+						33,832,516
19	0000977	Up in high-HIF1A PC tissues		qRT-PCR												31,402,756
20	0071036	Up	MEL	qRT-PCR				+				PET-CT SUVmax value	0.65	OS	+	33,507,122
21	0000677	Up	MEL	qRT-PCR				+		+	+			OS	+	33,413,045
22	0000069	Up		qRT-PCR	+				+				0.8944			33,324,055
23	0,060,055	Up		qRT-PCR						+			0.9093			33,469,368
24	0036627	Up	MEL	qRT-PCR			+	+		+				OS	+	29,709,702
25	0001460	Up	MEL	qRT-PCR							+			OS	+	33,750,389
26	0007534	Up	MEL	qRT-PCR			+	+						OS	+	30,382,592
27	IARS	Up	MEL	qRT-PCR					+	+	+			OS	+	30,064,461
28	0030235	Up		qRT-PCR	+			+						OS	+	30,591,218
29	ASH2L	Up		qRT-PCR				+		+				OS	+	31,718,694
30	0001013	Up	MEL	qRT-PCR		+		+	+	+				OS		33,563,550
31	ADAM9	Up		qRT-PCR				+		+				OS		31,810,373
32	0000284	Up		qRT-PCR								GEM resistance		OS		32,104,074
33	LDLRAD3	Up		qRT-PCR										OS		31,521,692
34	0001568	Up		qRT-PCR and FISH										OS		32,193,152
35	03955	Up		qRT-PCR										OS		33,864,618
36	0009065	Up		qRT-PCR						+				OS/DFS	+	32,375,768
37	0092314	Up	MEL	qRT-PCR		+		+		+				OS/DFS		33,842,379
38	0005273	Up		qRT-PCR				+	+					OS/PFS		33,275,224
39	0001649	Down	MV	qRT-PCR		+	+							OS	+	29,969,694
40	0000979	Down	MEL	qRT-PCR		+		+						OS		32,878,470
41	0086375	Down		qRT-PCR				+		+				OS/DFS	+	32,366,257

MEL: Median Expression Level; MV: Mean Value; GEM: Gemcitabine; AUC: Area Under the Curve; OS: Overall Survival; DFS: Disease-free Survival; PFS: Progression-free survival

**Table 5:** Overview of prognostic, diagnostic and clinicopathological significance of circRNAs in pancreatic cancer plasmas

No	Circ	Expression	Cut-off	Detect	Clinical significance	AUC	Sensitivity	Specificity	Survival	Prognostic biomarker	Reference
1	0002130/0000896/0101692/0005882/0001250/0000128	Up		qRT-PCR							31,605,569
2	0103896/0006662/0035432/0094190	Down		qRT-PCR							31,605,569
3	0,001,013	Up		qRT-PCR							33,563,550
4	chr12:74,678,804–74,700,449	Up		qRT-PCR							32,879,441
5	0006988	Up		qRT-PCR	CA19-9 level, Vessel invasion, Lymphatic invasion, Distance metastasis, TNM stage	0.67	0.5738	0.7049			29,307,994
6	0000677	Up		qRT-PCR		0.716	0.6276	0.7429			33,413,045
7	0036627		MEL	qRT-PCR	Duodenal invasion, Vessel invasion, T stage, TNM stage				OS	+	29,709,702
8	IARS	Up		qRT-PCR	Exosomes from patients with or without metastasis						30,064,461
9	chr14:101,402,109–101,464,448/chr4:52,729,603–52,780,244	Up in non-GEM-responsive		qRT-PCR							29,922,161

MEL: Median Expression Level; AUC: Area Under the Curve; OS: Overall Survival

### Diagnostic biomarkers for PC

Increasing research suggests that circRNAs might serve as potential diagnostic biomarkers for PC. Han et al. found that the AUC of hsa_circ_0071036 for detecting PC was 0.65, suggesting its value in PC diagnosis [47]. In addition, this circRNA was correlated with lymphatic invasion and was an independent risk factor for prognosis. Shen et al*.* found that hsa_circ_0000677 was highly expressed in PC tissue and plasma, and its AUC in plasma was 0.716 (sensitivity = 0.6276, specificity = 0.7429) [75]. Ye et al*.* found that the AUC of hsa_circ_0000069 in the diagnosis of PC was 0.894 and correlated with tumor size and distant metastasis [124]. Zhang et al. found that the AUC of hsa_circ_0060055 was 0.9093, indicating its extremely reliable diagnostic value [80].

### Prognostic biomarkers for PC

In our data collection, we found that circRNAs were strongly associated with PC prognosis. Li et al. found that hsa_circ_0036627 expression was correlated with TNM stage and was positively correlated with overall survival (OS) in PC patients [59]. Similar effects of hsa_circ_0036627 were found in PC plasma; this circRNA was correlated with TNM stage, vessel invasion and patient prognosis. Therefore, hsa_circ_0036627 could be a stronger indicator of patient prognosis than many other circRNAs. Fourteen circRNAs were reported to be positively correlated with the prognosis of PC patients, while 3 circRNAs were reported to have the opposite effects. Among them, hsa_circ_0009065, hsa_circ_0092314 and hsa_circ_0086375 were associated with disease-free survival (DFS), and hsa_circ_0005273 was associated with progression-free survival (PFS) [50, 63, 88, 107]. Some circRNAs associated with PC may also serve as independent prognostic factors. Using Cox regression, Li et al*.* identified hsa_circ_0036627 as an independent risk factor for prognosis in both PC tissues and plasma samples [59]. In addition, 8 circRNAs have been reported as independent risk factors in PC.

## Future perspective

Considering that circRNAs play indispensable roles in tumor pathogenesis, it is of great significance to design potential diagnostic and therapeutic strategies targeting circRNAs to gain control of malignant tumors, such as pancreatic cancer.

One feasible method is to interfere with circRNA expression using siRNAs or DNA plasmids, which is the most widely used method in basic research to regulate ncRNAs [125, 126]. To the best of our knowledge, there is no specific literature reporting clinical trials of circRNA-related therapies focusing on PC. In 2009, Mizrahi A et al. designed a DNA plasmid called H19-DTA, containing the diphtheria toxin-A gene, to target long noncoding RNA H19 (lncRNA H19, which is a ncRNA) expression. In vivo experiments showed that H19-DTA was able to suppress the growth of multiple cancer types [127]. Later, two clinical trials were conducted to verify the efficacy of H19-DTA in cancer patients, and both studies showed suppression of tumor growth and a prolonged survival time [128, 129]. Although clinical applications are still some way off, in the future, circRNAs might have clinical efficacy similar to lncRNA H19.

Recently, the development of clustered regulatory interspaced short palindromic repeats/CRISPR-associated protein 9 (CRISPR-Cas9) technology and its use in a variety of diseases have drawn much attention [130]. Despite the limitations in this field, ncRNA editing using CRISPR-Cas9 technology has been explored in various cancer types. Li et al. demonstrated that knockout of Fli1 exonic circRNAs using CRISPR/Cas9 technology significantly inhibited the migration and metastasis ability of non-small-cell lung cancer cells compared to short hairpin RNA (shRNA)-mediated knockdown [131]. In addition, Zhen et al. found that silencing lncRNA UCA1 via the CRISPR/Cas9 method effectively blocked the progression of bladder cancer [132]. Notably, lncRNA UCA1 is also highly expressed in PC, and downregulation of UCA1 effectively suppresses PC cell proliferation, promotes apoptosis and induces cell cycle arrest [133]. These clues prompted us to reflect on the possibility of inhibiting UCA1 expression via CRISPR/Cas9 technology to treat PC. To date, no investigation using CRISPR-Cas9-mediated circRNA editing has been reported in PC, but in the near future, this approach could become a promising strategy leading to effective PC treatments.

Strategies for safely, efficiently and continuously transporting stably altered circRNAs to target cells or organs are also needed for the future application of circRNAs. In recent years, exogenous nanoparticles, which can act as carriers for novel genes and drugs, have attracted wide attention [134]. Compared with traditional treatments, nanoparticles can reduce the concentration of a drug needed to induce effects that are otherwise only achieved with a high drug or radiation dose while increasing drug distribution in target organs and avoiding systemic damage [135]. Another emerging approach for targeting circRNAs is exosomes, which are defined as microvesicles with diameters of 30–100 nm that can be released from cells to exert intercellular communication functions [136]. Exosomes containing circRNAs have been shown to regulate the metastasis of PC cells [85]. Moreover, exosomes naturally exist in the body; thus, they have better histocompatibility than nanoparticles [137]. Despite the tremendous progress made in these areas, research findings are still theoretical, and no treatment based on nanoparticles or exosomes has yet been approved in the clinic.

Early diagnosis of PC is important for improving the 5-year survival rate [138]. A large number of studies have shown that circRNAs can serve as ideal noninvasive biomarkers for PC diagnosis and prognosis determination. In the future, a gold standard circRNA detection method should be identified to standardize the detection of circRNAs in various laboratories. In addition, multicenter, multipopulation trials with large sample sizes should be carried out to obtain more clinically significant thresholds. In practice, it should be noted that PC development is a long-term and chronic process. The potential role of circRNAs in the early diagnosis of precancerous lesions, including pancreatic intraepithelial neoplasia (PanIN), IPMN, and mucinous cystadenoma (MCN), should be emphasized. Finally, noninvasive or minimally invasive detection methods are the ultimate goal. Trials related to circRNA detection in peripheral blood and endoscopic biopsy samples (pancreatic juice or tissue) should be carried out in the early stages of clinical research.

## Conclusions

Despite the extensive efforts made in recent years in surgery- and chemoradiotherapy-based PC treatment, PC remains the seventh deadliest cancer worldwide; thus, better biomarkers and therapeutic strategies are needed for PC in clinical practice. With the help of new detection technologies, studies focusing on circRNAs have become a hotspot in the field of biological science, especially in the study of diverse cancer type. In this review, we comprehensively summarized the biogenesis mechanisms, classifications and modes of action of circRNAs and reviewed the functions and mechanisms of circRNAs in PC. Additionally, the clinical significance of circRNAs in PC was discussed. However, what has been revealed is only the tip of the iceberg, and there are still several obstacles along the road to a thorough understanding of the roles of circRNAs in PC. For example, the majority of recent studies have focused on the ceRNA function of circRNAs, whereas the interactions between circRNAs and other molecules, especially interactions among circRNAs themselves, have rarely been reported. Additionally, to date, no circRNAs have been approved for the diagnosis or treatment of PC. Therefore, there is still a long way to go before these findings can be translated from bench to the bedside. Nevertheless, with the continued emergence of more gratifying investigations, we believe that this will happen in the near future.

## Data Availability

All data generated or analyzed during this study are included in this published article.

## Abbreviations

PC: Pancreatic cancer
circRNA: Circular RNA
mFOLFIRINOX: Modified FOLFIRINOX
pre-mRNA: Precursor mRNA
EcRNA: Exonic circRNA
EIciRNA: Exon–intron circRNA
ciRNA: Circular intronic RNA
RBP: Rna binding protein
QKI: Quaking
EMT: Epithelial-mesenchymal transition
MBL: Muscleblind
ADAR: Adenosine deaminase acting on RNA
A-to-I: Adenosine-to-inosine
DHX9: Dexh-box helicase 9
miRNA: MicroRNA
Pol II: Rna polymerase II
snRNP: U1 small nuclear ribonucleic proteins
OFR: Open reading frame
IRES: Internal ribosome entry site
m6A: N6-methyladenosine
YTHDF3: Yt521-b homology domain-containing family 3
EIF4G2: Eukaryotic translation initiation factor 4 gamma 2
ceRNA: Competing endogenous RNA
MRE: MiRNA response element
p21: Cyclin-dependent kinase inhibitor 1
CDK2: Cyclin-dependent kinase 2
ID1: Inhibitor of differentiation 1
E2F1: E2f transcription factor 1
HIF1α: Tumor-related proteins hypoxia inducible factor 1 alpha
FAK: Focal adhesion kinase
MDM2: Mouse double minute 2
rRNA: Ribosome RNA
BSJ: Back splicing junction
RO: Reverse overlap
EGFR: Epidermal growth factor receptor
GLI1: Glioma-associated oncogene 1
SUFU: Suppressor of fused
BTG2: B-cell translocation gene 2
RhoA: Ras homolog family member A
RIP: Rna-binding protein immunoprecipitation
MACC1: Metastasis associated in colon cancer 1
KLF5: Kruppel-like factor 5
HOXC6: Human homeobox C6
MET: Mesenchymal-epithelial transition
MMP: Matrix metalloproteinases
NKG2D: Natural killer group 2 member d
mMICA: Membrane-bound mhc class i polypeptide-related sequence a
ZEB1: Zinc finger E-box-binding protein 1
TAM: Tumor-associated macrophages
YBX1: Y-box binding protein 1
hnRNPK: Heterogeneous nuclear ribonucleoprotein K
NUF2: Ndc80 kinetochore complex component
PDXK: Pyridoxal kinase
CT: Computer tomography
FDA: Food and drug administration
ctDNA: Circulating tumor DNA
CTC: Circulating tumor cells
IPMN: Intraductal papillary mucinous neoplasm
AUC: Area under the ROC curve
OS: Overall survival
DFS: Disease-free survival
PFS: Progression-free survival
CRISPR-Cas9: Clustered regulatory interspaced short palindromic repeats/CRISPR-associated protein 9
shRNA: Short hairpin RNA
ncRNA: Noncoding RNA
lncRNA: Long noncoding RNA
PanIN: Pancreatic intraepithelial neoplasia
MCN: Mucinus cystadenoma
